# Longitudinal Single‐Cell Transcriptomic Profiling Reveals Dynamic Immune Cell Alterations During Burosumab Therapy in X‐Linked Hypophosphatemia

**DOI:** 10.1002/pdi3.70053

**Published:** 2026-06-27

**Authors:** Yue Xie, Li Li, Rong Li, Yihong Sun, Ting Zhou, Yupeng Cun, Gaohui Zhu

**Affiliations:** ^1^ Chongqing Key Laboratory of Child Neurodevelopment and Cognitive Disorders Pediatric Research Institute, Children's Hospital of Chongqing Medical University Chongqing China; ^2^ Ministry of Education Key Laboratory of Child Development and Disorders, National Clinical Research Center for Child Health and Disorders Children's Hospital of Chongqing Medical University Chongqing China; ^3^ Department of Endocrinology, Chongqing Key Laboratory of Child Rare Diseases in Infection and Immunity Children's Hospital of Chongqing Medical University Chongqing China

**Keywords:** Burosumab, longitudinal study, osteoclast differentiation, single‐cell RNA sequencing, X‐linked hypophosphatemia

## Abstract

X‐linked hypophosphatemia (XLH) is a rare hereditary disorder characterized by *PHEX* gene mutations, elevated *FGF23* levels, and impaired bone mineralization. Burosumab, a monoclonal antibody targeting *FGF23*, has demonstrated clinical efficacy; however, the immunological dynamics during treatment remain unexplored. This study employed longitudinal single‐cell RNA sequencing (scRNA‐seq) to characterize peripheral blood immune cell alterations across multiple treatment stages in pediatric XLH. We performed scRNA‐seq on peripheral blood mononuclear cells from pediatric patients with XLH at five time points spanning pretreatment and burosumab therapy phases, along with healthy pediatric controls. A total of 93,112 cells were analyzed using comprehensive bioinformatic pipelines, including unsupervised clustering, pseudotime trajectory analysis, temporal gene expression profiling, and cell–cell communication inference. Eleven major immune cell populations were identified, with notable dynamic alterations in T cells and natural killer (NK) cell subtypes across treatment stages. The cellular proportion of T helper 2 (Th2) cells and regulatory T (Treg) cells were elevated before treatment and normalized during therapy, whereas T helper 17 (Th17) cells exhibited reciprocal patterns. Genes upregulated in Treg cells during early treatment were enriched in osteoclast differentiation pathway. Natural killer subtype 2 cells showed enrichment in osteoclast differentiation and interleukin‐12 response pathways. Cell–cell communication analysis identified dynamic interactions among Th2 cells, Th17 cells, Treg cells, and NK cell subtypes mediated by KLRB1–CLEC2D and SELL–SELPLG ligand‐receptor pairs. This longitudinal transcriptomic study provides the first comprehensive characterization of peripheral immune dynamics during burosumab therapy in XLH, offering new insights into the immunological mechanisms underlying treatment response.

## Graphical abstract

This longitudinal single‐cell study reveals dynamic immune remodeling during burosumab therapy in pediatric X‐linked hypophosphatemia (XLH), highlighting coordinated changes in T cell subtypes (Th2, Th17, and Treg) and natural killer cell subtypes (NK2 and NK3) and immune pathways linked to osteoclast differentiation and treatment response.

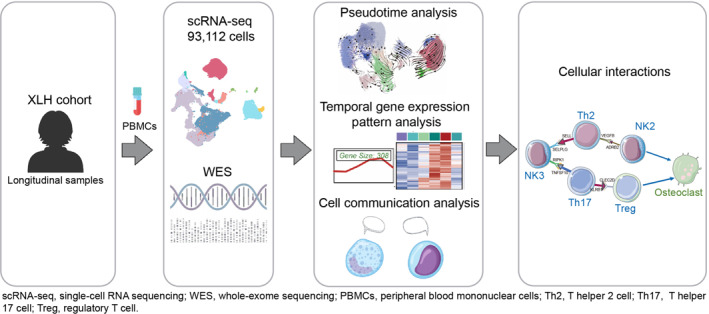

## 1 Introduction

X‐linked hypophosphatemic (XLH) rickets is an inherited and rare disease caused by mutations in phosphate‐regulating endopeptidase homologous X‐linked (*PHEX*) gene, which follows an X‐linked dominant inheritance pattern. XLH represents the most common form of hereditary hypophosphatemia, accounting for approximately 90% of familial and 70% of sporadic cases, with an estimated prevalence of 3.9–5.0 per 100,000 individuals [1, 2]. Impaired phosphate reabsorption in renal tubules, due to tubular injury or genetic defects in sodium–phosphate cotransporters, leads to hypophosphatemia. Persistent phosphate loss in children results in defective bone mineralization, skeletal deformities, growth retardation, and elevated fibroblast growth factor 23 (*FGF23*) signaling [3]. Mutations in *PHEX, FGF23, DMP1, ENPP1*, and *SLC34A3* underlie various forms of hypophosphatemic rickets, exhibiting genetic and functional heterogeneity [3, 4, 5]. *PHEX* mutations elevate circulating *FGF23*, suppressing phosphate reabsorption and causing hypophosphatemia. Conventional therapy with phosphate and active vitamin D provides limited efficacy. Burosumab, a fully human monoclonal antibody targeting *FGF23*, effectively restores phosphate balance, enhances bone mineralization, and improves growth outcomes [6, 7]. Despite the clinical success of Burosumab, the immunological responses during treatment and their potential relationships to bone metabolism remain poorly understood.

Single‐cell RNA sequencing (scRNA‐seq) has transformed the understanding of cellular heterogeneity and transcriptional dynamics, facilitating rare disease research by identifying novel biomarkers and therapeutic targets [8, 9, 10]. Previous scRNA‐seq studies identified *FGF23*‐expressing osteocytes in mice, highlighting osteocyte heterogeneity and the molecular basis of XLH [11]. However, the immune landscape and treatment‐associated cellular changes in patients with XLH remain unexplored at the single‐cell level.

In this study, we conducted a longitudinal single‐cell transcriptomic analysis of peripheral blood mononuclear cells (PBMCs) from pediatric patients with XLH across multiple treatment stages. By profiling over 93,000 single cells, we identified dynamic alterations in T cells and natural killer (NK) cell subtypes and uncovered potential cellular interactions relevant to bone metabolism. This discovery‐driven approach provides novel insights into the immunological landscape of XLH during burosumab therapy and identifies candidate cellular targets for future mechanistic investigations.

## 2 Materials and Methods

### 2.1 Patients and Study Design

This longitudinal exploratory study was designed to characterize immune cell dynamics during burosumab therapy. Given the ultrarare nature of XLH (estimated prevalence, 3.9–5.0 per 100,000 individuals) [1, 2], we employed an intensive sampling strategy focusing on deep single‐cell profiling rather than large cohort enrollment. PBMCs were collected from one patient with XLH before initial burosumab therapy (XLH_0), and one patient with XLH at four logitudinal time points during burosumab therapy. Burosumab was administered every 2 weeks, and samples were collected following the first, second, third, and fourth doses (XLH_1.1, XLH_1.2, XLH_1.3, XLH_1.4). Patient received oral vitamin D supplementation (800–1,000 IU/d) as part of his standard treatment regimen. None of the participants had evidence of acute or chronic infectious disease at the time of sample collection. Control scRNA‐seq data from four healthy pediatric subjects were obtained from a published dataset [12] and were designated as the Normal group for subsequent analysis. For comparative analysis, samples were categorized into four study subgroups: Normal (healthy control), Pre_treatment (XLH_0), Early_stage (XLH_1.1 and XLH_1.2), and Late_stage (XLH_1.3 and XLH_1.4). To facilitate downstream differential expression and comparative analyses, samples from the Early_stage and Late_stage groups were collectively designated as the Treatment_stage group. All human procedures were approved by the Ethics Committee of the Children's Hospital of Chongqing Medical University (NO. 2022340), with written informed consent obtained from the patient's parents.

### 2.2 PBMCs Isolation, Single‐Cell Library Construction, and Sequencing

PBMCs were isolated from 3 mL of ethylenediaminetetraacetic acid (EDTA)‐treated blood using Ficoll‐Paque PREMIUM, following the manufacturer's protocol. Cell suspensions were pooled, diluted to 1 × 10^6^ cells/mL, and loaded onto the 10× Genomics platform for cDNA library construction. Libraries were sequenced on the Illumina NovaSeq 6000 platform using paired‐end sequencing. Cryopreserved samples were thawed and processed immediately using the 10× Genomics  Chromium Single Cell 3′ Reagent Kit v3, following the manufacturer's guidelines.

### 2.3 Data Processing, Integration, and Cluster Annotation

Raw base call (BCL) files were demultiplexed and aligned to the human reference genome (GRCh38) using Cell Ranger (v1.1.0) [13]. Doublets were removed using DoubletFinder (v2.0.3) [14], and data were normalized and processed in Seurat (v4.3.0) [15]. Cells with over 3,000 genes, fewer than 200 genes, or more than 15% mitochondrial counts were excluded. A total of 93,112 high‐quality cells were retained for downstream analysis, comprising approximately 70,000 cells from XLH patients and 23,000 cells from healthy controls. To minimize potential batch effects between in‐ house and public datasets, we applied harmony integration after standard Seurat processing. Following NormalizeData method, the top 3,000 highly variable genes were selected for dimensionality reduction. Principal component analysis (PCA) and uniform manifold approximation and projection (UMAP) were used for dimensionality reduction, and clusters were identified by the Wilcoxon rank‐sum test (|log2 fold change| ≥ 2 and adjusted *p* ≤ 0.05).

### 2.4 Functional Enrichment Analysis and Differential Expression Analysis

Kyoto Encyclopedia of Genes and Genomes (KEGG) and Gene Otology (GO) [16, 17] enrichment analyses and differential expression analysis were performed using ClusterGVis (v0.1.2) (v0.1.2, https://github.com/junjunlab/ClusterGVis). Differentially expressed genes (DEGs) among NK cell subtypes were identified with adjusted *p* < 0.05 and |log2 fold change| > 0.25. DEGs were subsequently subjected to KEGG and GO enrichment analyses. DEGs among the Pre_treatment and Normal groups as well as the Treatment_stage and Pre_treatment groups were identified using the FindMarkers function in Seurat, with gene showing |log2 fold change| > 0.1 and adjusted *p* value < 0.05 considered significant.

### 2.5 Cell–Cell Communication Analysis

CellPhoneDB (v3.0.0) [18] was used to infer molecular interactions between cell types based on ligand‐receptor pairs (*p* < 0.05). This analysis identified significant cell–cell communication networks.

### 2.6 Single‐Cell Trajectory Analyses

Monocle2(v2.18.0) [19] was used to order cells along a pseudotime trajectory, reflecting their progression through developmental programs. Trajectory inference was performed using Monocle2 with the DDRTree algorithm. Cells were ordered along pseudotime following dimensionality reduction and trajectory reconstruction, and the root state was automatically defined based on the reconstructed topology. Pseudotime represents an inferred ordering of cellular transcriptional states, which is different from sampling time.

To complement Monocle2‐based trajectory reconstruction, we used scVelo (v0.2.4) as an independent method to assess lineage transition directionality by testing whether the inferred RNA velocity field was concordant with the Monocle2 trajectory direction. Within the myeloid and B‐cell lineages, cells were ordered along Monocle2‐inferred pseudotime trajectory. Pseudotime‐dependent genes identified by Monocle2 were used to generate the pseudotime heatmap. Gene expression values were log‐normalized and scaled (*z*‐scored) per gene, and genes were clustered into modules based on similar expression dynamics along pseudotime.

### 2.7 Network Analysis of *PHEX* Related Genes

A *PHEX‐*related protein–protein interaction (PPI) network was constructed using STRING (V12.0) [20] with a confidence interaction score of 0.15. The max number of interactions in both the first and second shells was set to 5. The constructed PPI network consisted of 23 gene nodes and 328 interactions. The network was visualized using Gephi [21], and a minimum spanning tree [22, 23] was used to identify hub genes.

### 2.8 Temporal Gene Expression Pattern Analysis

Mfuzz (v2.68.0) [24] was used to analyze temporal gene expression patterns across all study groups. Genes showing significant expression changes across all study groups (defined as |log2 fold change| > 1 and adjusted *p* value < 0.05) were selected for Mfuzz clusterting. Selected genes were clustered into temporal expression patterns according to their expression dynamics, and functional enrichment was performed using clusterProfile (v4.16.0) [25].

### 2.9 Whole Exome Sequencing (WES) Analysis

WES data were aligned to the human reference genome (hg38) using BWA–MEM (v0.7.17) [26] with default parameters. BAM files were sorted according to the genome analysis toolkit (GATK) best practices, polymerase chain reaction duplicates were removed, and base quality recalibration was performed. Germline variants were called using GATK HaplotypeCaller (v4.2), followed by variant quality score recalibration (VQSR). Variants passing VQSR filtering with tranche sensitive thresholds of 99.80 for single nucleotide polymorphisms and 99.70 for insertations/delections were retained for downstream analysis.

### 2.10 Copy Number Variation (CNV) Analysis

CNV profiles were inferred from scRNA‐seq data using the CopyKAT (v1.0.4) [27] R package. Raw gene expression count matrices were used as input, and default parameters were applied unless otherwise specified. The inferred CNV profiles were subsequently used to measure cell division activity in all study groups.

### 2.11 Association Analysis Between Cell Subtype Features, Metabolic Pathway Activities and Clinical Data

AUCell (v1.30.1) [28] was used to calculate the AUCell score of the top 20 highly expressed genes in each cell subtype. Mean AUCell value was used as the cell feature value of each subtype. Locally estimated scatterplot smoothing (LOESS) and regression was applied to fit the relationship between cell features, cell frequency, and clinical laboratory parameters, including serum phosphate, total serum calcium, alkaline phosphatase, and vitamin D. Metabolic pathway activities were inferred using METAFlux (v1.0.0) based on scRNA‐seq data. Flux scores were calculated for each cell subtype across each study group using the default parameters. The Vitamin D metabolism pathway was extracted for downstream analysis, and its activity was compared across all study groups in different cell subtypes.

## 3 Results

### 3.1 Longitudinal Single‐Cell Profiling Reveals Dynamic Immune Cell Composition During XLH Treatment

**FIGURE 1:**
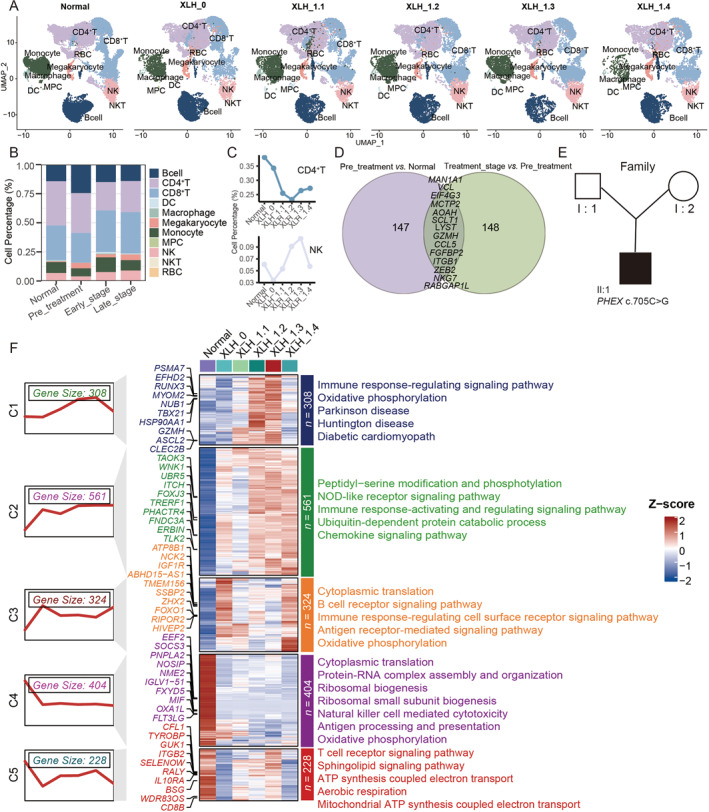
Landscape of peripheral blood cells of X‐linked hypophosphatemia (XLH) patients across monoclonal antibody treatment. (A) Uniform manifold approximation and projection (UMAP) of 93,112 cells colored by cell type and split by group. (B) Histogram illustrating the percentage of different cell types across four study subgroups: Normal (healthy control), Pre_treatment (XLH_0), Early_stage (XLH_1.1 and XLH_1.2), and Late_stage (XLH_1.3 and XLH_1.4). (C) Line graph displaying variation trends of cell percentages along study groups in CD4^+^ T cells and natural killer (NK) cells. (D) Venn plot showing upregulated genes both in the Pre_treatment group (XLH_0) versus Normal group and Treatment_stage groups (including the Early_stage and Late_stage) versus Pre_treatment group. Numbers in the Venn plot represent the number of differentially expressed genes in each comparison. (E) Pedigree chart showing *PHEX* gene mutation of Patient 1's family. Patient 1 (II:1) carries a *PHEX* c.705C>G variant inherited from his mother (I:2). (F) Heatmap showing genes and Gene Ontology terms and Kyoto Encyclopedia of Genes and Genomes pathways in different temporal gene expression patterns over study groups. Line graph on the left shows trends over study groups. The number shown in each panel (Gene Size) indicates the number of genes assigned to the corresponding temporal expression pattern. Normal represents healthy controls, XLH_0 denotes pre‐treatment sample from one patient with XLH, whereas XLH_1.1‐XLH_1.4 denote logitudinal samples collected from the second patient with XLH during burosumab treatment. ATP, adenosine triphosphate; Bcell, B cell; CD4^+^T, CD4^+^ T cell; CD8^+^T, CD8^+^ T cell; DC, dentritic cell; MPC, multipotent progenitor cell; NKT, natural killer T cell; NOD, nucleotide‐binding oligomerization domain; RBC, red blood cell.

To comprehensively characterize peripheral blood immune dynamics during XLH treatment, we performed scRNA‐seq on PBMCs collected from pediatric patients with XLH, including a pre‐treatment patient (XLH_0) and a logitudinally treated patient (XLH_1.1, XLH_1.2, XLH_1.3, and XLH_1.4). Publicly available scRNA‐seq data from healthy pediatric donors were included as the Normal group. After stringent quality control, 93,112 cells were retained for analysis, representing one of the largest single‐cell datasets in hereditary hypophosphatemic disorders to date. Eleven cell clusters were identified, including B cells, CD4^+^ T cells, CD8^+^ T cells, macrophages, dendritic cells (DCs), megakaryocytes, monocytes, multipotent progenitor cells (MPCs), NK cells, natural killer T (NKT) cells, and red blood cells (RBCs) using canonical markers (Figure 1A, Figure S1A and S1B in Supporting Information S1).

To investigate cellular composition across different study groups, samples were categorized into four subgroups: Normal (healthy control), Pre_treatment (XLH_0), Early_stage (XLH_1.1 and XLH_1.2), and Late_stage (XLH_1.3 and XLH_1.4) (Figure 1B). The proportion of B cells increased in the Pre_treatment group, decreased during therapy, and reverted to normal levels in the Late_stage group (Figure 1B). The proportion of NK cells and CD8^+^ T cells were reduced in the Pre_treatment group but returned to normal levels in the Late_stage group (Figure 1B). In the following analysis, we focused on NK cells and CD4^+^ T cells associated with osteoclast differentiation. The longitudinal sampling design enabled us to capture the temporal trajectory of immune cell composition during treatment. Notably, the proportion of CD4^+^ T cells gradually decreased during treatment, reached its lowest point at the mid‐treatment stage (XLH_1.2), and rebounded in late stages (XLH_1.3 and XLH_1.4) (Figure 1C). The proportion of NK cells was reduced in the pre‐treatment group (XLH_0), peaked during treatment, and returned to normal levels in the late stage (XLH_1.4) (Figure 1C). Changes of the proportion of other cell types across the normal group, pre‐treatment group, and treatment groups are shown in Figure S1C in Supporting Information S1. Mutual DEGs between the Pre_treatment group (XLH_0) and the Normal group, as well as between the Treatment_stage group (including the Early_stage and Late_stage groups) and the Pre_tretment group, are shown in Figure 1D. These mutual DEGs may represent treatment‐responsive genes whose abnormal expression in XLH was partially reversed during burosumab therapy. These include immune‐related genes (*GZMH*, *CCL5*, and *NKG7*), cytoskeleton‐associated genes (*VCL*, *LYST*, and *ITGB1*), and genes involved in fatty acid and purine metabolism (*AOAH* and *SCLT1*) (Figure 1D, Table S1 in Supporting Information S3). WES analysis of the XLH proband and his parents identified a germline *PHEX* variant, c705C>G, in the proband, which was inherited from his mother (Figure 1E).

To further investigate dynamic gene expression changes during treatment, all genes were classified into five temporal expression patterns (C1–C5) using Mfuzz (Figure 1F, Table S2 in Supporting Information S3). The C5 pattern comprised 228 genes whose expression decreased in the pre‐treatment group and returned to normal levels during treatment, while decreased again in the late stage. GO and KEGG enrichment analyses showed that these genes were primarily involved in adenosine triphosphate (ATP) synthesis coupled with electron transport, T cell receptor signaling pathway, and the sphingolipid signaling pathway (Figure 1F, Table S2 in Supporting Information S3). Genes in the C1 pattern showed increased expression during the mid‐treatment stage, followed by a reduction to near‐normal levels in the late stage. These genes were mainly involved in immune response–regulating signaling pathway and oxidative phosphorylation (Figure 1F, Table S2 in Supporting Information S3). CNV profile inferred by the CopyKAT was used to evaluate cell division activity in peripheral immune cells. Aneuploid cells, defined as proliferative cells, were enriched in the early treatment stage (XLH_1.1), gradually decreased to near‐normal levels at the XLH_1.3 stage, and increased again at the XLH_1.4 stage (Figure S1D in Supporting Information S1), suggesting treatment‐associated immune remodeling and potential immune reactivation during later stages.

Altogether, we delineated the PBMC landscape of patients with XLH undergoing burosumab treatment, revealing dynamic immune and gene expression changes that could reflect disease progression and therapeutic response.

### 3.2 Functions of T Cell Subtypes Across Treatment Time Points

**FIGURE 2:**
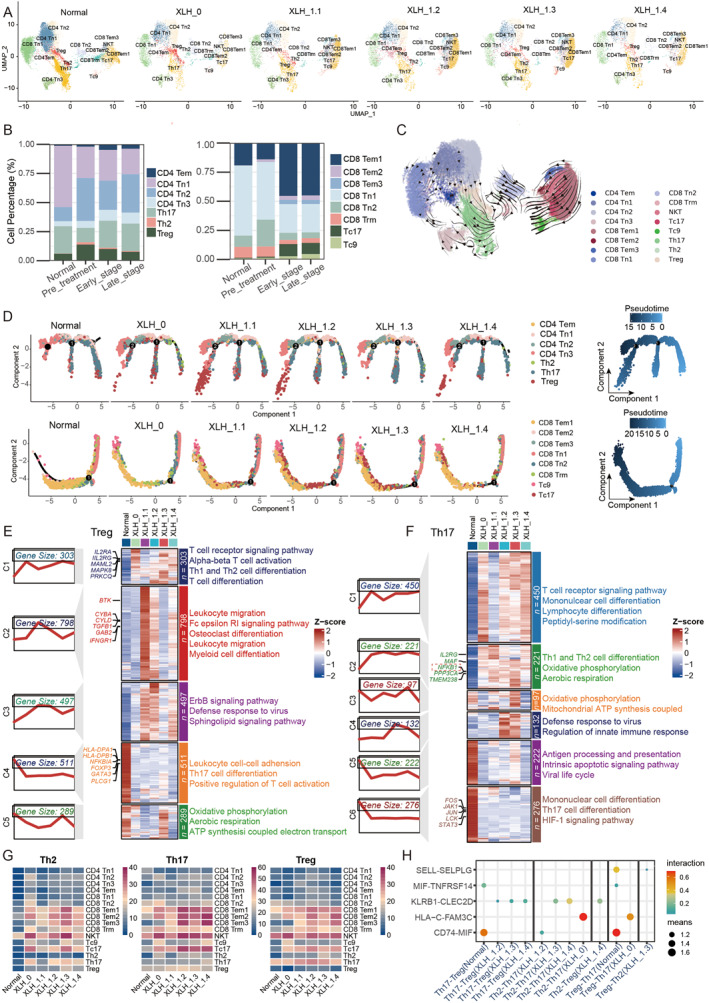
T cell subtype analysis in different treatment time points. (A) Uniform manifold approximation and projection (UMAP) plot of 54,959 T cells colored by cell subtype and split by group. (B) Histogram illustrating the percentage of CD4^+^ and CD8^+^ T cell subtypes across four study subgroups: Normal (healthy control), Pre_treatment (XLH_0), Early_stage (XLH_1.1 and XLH_1.2), and Late_ stage (XLH_1.3 and XLH_1.4). (C) UMAP plot illustrating differential trajectories of T cell subtypes analyzed using scVelo. (D) UMAP plot illustrating the pseudotime trajectory of CD4^+^ T and CD8^+^ T cell subtypes in each group analyzed by monocle2. (E) Heatmap showing genes and Gene Ontology (GO) terms and Kyoto Encyclopeia of Genes and Genomes (KEGG) pathways in different temporal gene expression patterns over study groups in regulatory T (Treg) cells. Line graph on the left shows trends over study groups. The number shown in each panel (Gene Size) indicates the number of genes assigned to the corresponding temporal expression pattern. (F) Heatmap showing genes and GO terms and KEGG pathways in different temporal gene expression patterns over study groups in T helper 17 (Th17) cells. (G) Heatmap showing cell–cell communication numbers of Th17, T helper 2 (Th2), and Treg cells with other T cell subtypes. (H) Dot plot of the predicted interaction pairs between Th2, Th17, and Treg cells. The color indicates interaction intensity, the dot size indicates the mean communication probability. For ligand‐receptor pairs, the first molecule is the ligand and the second is the receptor; for cell pairs, the first cell type is the sendor and the second is the receiver. ATP, adenosine triphosphate; CD4 Tem, memory CD4^+^ T cell; CD4 Tn1‐CD4 Tn3, naive CD4^+^ T‐cell subtypes; CD8 Tem1‐CD8 Tem3, memory CD8^+^ T‐cells subtypes; CD8 Tn1‐CD8 Tn2, naive CD8^+^ T‐cell subtypes; CD8 Trm, tissue‐resident memory CD8^+^ T cell; HIF, hypoxia‐inducible factor; NKT, natural killer T cell; Tc9, interleukin‐9‐producing cytotoxic T cell; Tc17, interleukin‐17‐producing cytotoxic T cell.

T cells were classified into seven CD4^+^ T cell subtypes, eight CD8^+^ T cell subtypes, and NKT cells based on canonical markers (Figure 2A, and Figure S2A and S2B in Supporting Information S1). In the comparison across different study subgroups, the proportion of T helper 2 (Th2) cells and regulatory T (Treg) cells were elevated in the Pre_treatment group and gradually decreased in the Early_stage and Late_stage groups (Figure 2B). In contrast, the proportion of T helper 17 (Th17) cells was reduced in the Pre_treatment group and returned to normal levels in the Early_stage and Late_stage groups (Figure 2B). Specifically, the proportion of Th2 and Treg cells increased in the pre‐treatment group (XLH_0), with the proportion of Th2 cells showing a progressive decline from normal group through treatment time points (XLH_0 and XLH_1.1–XLH_1.4). Treg cells proportions decreased to their lowest level at the XLH_1.3 stage and slightly rebounded at the XLH_1.4 stage (Figure S2C in Supporting Information S1), whereas Th17 cells proportions exhibited a fluctuating trend (Figure S2C in Supporting Information S1). Pseudotime analysis results of the Monocle2 suggest a continuum of transcriptional states within the T‐cell compartment, where Treg, Th17, interleukin‐9‐producing cytotoxic T (Tc9) cells, and interleukin‐17‐producing cytotoxic T (Tc17) cells were enriched toward the terminal region of the inferred trajectory (Figure 2D). RNA velocity analysis using scVelo showed a transition direction consistent with the Monocle2‐inferred trajectory (Figure 2C). Here, pseudotime denotes a cellular state transition rather than actual treatment time.

Previous studies reported that Th17 cells promote osteoclast formation [29], and that Th17 and Treg cells are modulated by 1,25‐(OH)_2_D_3_ [30], suggesting that these two cell types may be associated with bone osteogenesis. Therefore, we further investigated whether Th17 and Treg cells in our data are associated with bone formation. Mfuzz clustering identified five temporal expression patterns (C1–C5) in Treg cells and six temporal expression patterns (C1–C6) in Th17 cells (Figure 2E,F; and Tables S3 and S4 in Supporting Information S3). Genes in the C2 temporal expression pattern of Treg cells, including *BTK*, *CYBA*, *CYLD*, and *TGFB1*, showed peak expression during early treatment and were enriched in the osteoclast differentiation pathway (Figure 2E, and Table S3 in Supporting Information S3), suggesting that their early upregulation may modulate osteoclast activity throughout therapy. Genes in the C1 temporal expression pattern of Treg cells, such as *IL2RA*, *IL2RG*, *MAML2*, and *MAPK8*, exhibited fluctuating expression and were enriched in Alpha‐beta T cell activation and T helper 1 (Th1) and Th2 cell differentiation (Figure 2E, and Table S3 in Supporting Information S3). In Th17 cells, *IL2RG*, *MAF*, and *NFKB1* were classified into the C2 pattern and were enriched in Th1 and Th2 cell differentiation, whereas genes in the C6 pattern were enriched in Th17 cell differentiation and highly expressed only in the normal group (Figure 2F, and Table S4 in Supporting Information S3). Additional Mfuzz analyses of CD4^+^ and CD8^+^ T cells are shown in Figure S2D and S2E of Supporting Information S1 (Table S5 in Supporting Information S3). Collectively, these results suggest that Treg, Th2, and Th17 cells may interact with each other and influence bone formation during treatment.

To further explore potential cellular communication, we analyzed interactions among Treg, Th2, and Th17 cells using CellPhoneDB (Figure 2G). Interactions between Th2 and Th17 cells were more frequent in disease groups (XLH_0 and XLH_1.2–XLH_1.4) than in the normal group, whereas interactions between Th17 and Treg cells were stronger during early treatment stages (XLH_1.1 and XLH_1.2) compared with the normal group and the late treatment stage (XLH_1.4) (Figure 2G). Ligand–receptor analysis showed that Th17–Treg interactions mainly occurred via KLRB1–CLEC2D pairs during treatment, whereas Th2–Th17 communicated through the same pairs in late stages (XLH_1.3 and XLH_1.4). Additionally, Treg–Th2 interactions were mediated by SELL–SELPLG pairs in the late treatment stage (XLH_1.3) (Figure 2H).

These findings reveal dynamic interactions among Treg, Th2, and Th17 cells during treatment, suggesting their potential roles in regulating bone formation across XLH treatment.

### 3.3 Dynamic Changing Patterns of NK Cell Subtypes Across Treatment

**FIGURE 3:**
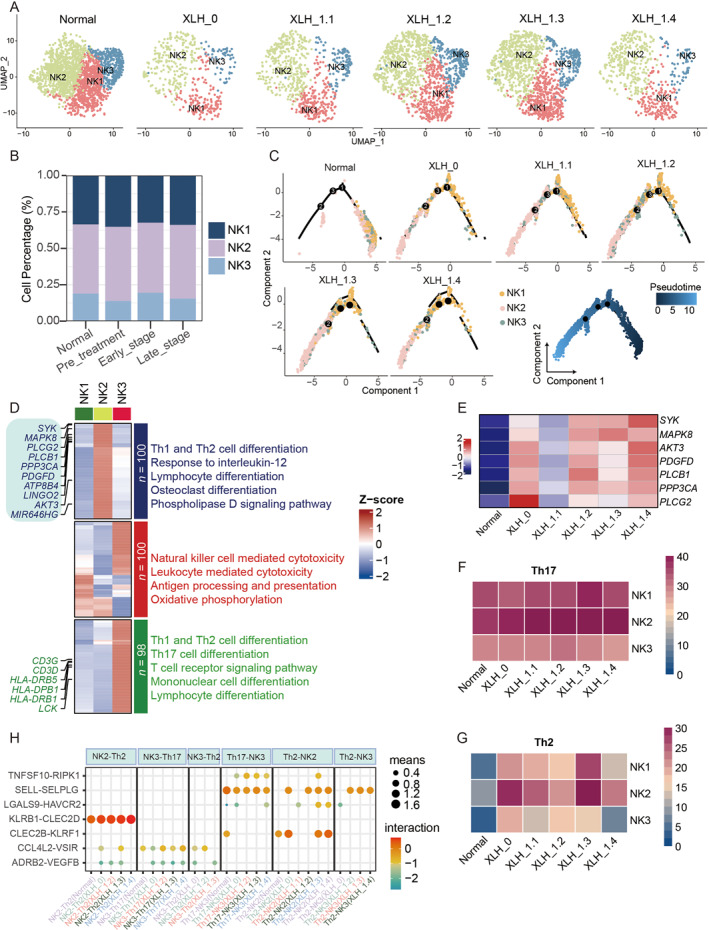
Changing patterns of natural killer (NK) cell subtypes during treatment. (A) Uniform manifold approximation and projection (UMAP) plot of 6,106 NK cells colored by cell subtype and split by group. (B) Histogram illustrating the percentage of NK cell subtypes across four study subgroups: Normal (healthy control), Pre_treatment (XLH_0), Early_stage (XLH_1.1 and XLH_1.2), and Late_stage (XLH_1.3 and XLH_1.4). (C) UMAP plot illustrating the pseudotime trajectory of NK cell subtypes in each group analyzed by monocle2. (D) Heatmap showing differential expression genes and relevant Kyoto Encyclopedia for Genes and Genomes pathways and Gene Ontology terms of each NK cell subtypes. Genes highlighted in blue box are osteoclast differentiation‐related genes identified through enrichment analysis and were used to generate the heatmap in (E). (E) Heatmap illustrating expression levels of genes related to the osteoclast differentiation pathway and expressed in NK2 cells in different study groups. (F) Heatmap showing cell–cell communication numbers of Th17 cells with NK cell subtypes. (G) Heatmap showing cell–cell communication numbers of Th2 cells with NK cell subtypes. (H) Dot plot of the predicted interaction pairs between NK2‐Th2, NK3‐Th17, NK3‐Th2, Th17‐NK3, Th2‐NK2, and Th2‐NK3. Interaction intensity was indicated by the color, and the mean communication probability was indicated by the dot size. In ligand‐receptor pairs, the first molecule is the ligand and the second is the receptor; in cell pairs, the first cell type is the sender and the second is the receiver. NK1–NK3, natural killer cell subtypes 1–3; Th1, T helper 1 cell; Th2, T helper 2 cell; Th17, T helper 17 cell.

A total of 6,106 NK cells were classified into three subtypes (NK1–NK3) based on known markers [31] (Figure 3A, and Figure S3A and S3C in Supporting Information S1). In the comparison across different study subgroups, the proportion of NK3 cells was lower in Pre treatment and Late_stage groups compared with the Normal and Early_stage groups (Figure 3B). Pesudotime trajectory analysis of NK cells placed NK1 cells toward the early region of the inferred trajectory (Figure 3C). NK1 cells of the normal group were relatively enriched in the early pseudotime region, whereas cells from treatment groups (XLH_1.1, XLH_1.2, XLH_1.3, and XLH_1.4) were predominantly distributed in the later pseudotime region than in the normal group, indicating a shift in NK‐cell transcriptional states across conditions rather than a direct measure of treatment duration. Along this inferred pesudotime trajectory, NK2 and NK3 cells were preferentially distributed toward later pseudotime regions, suggesting that these subsets occupy transcriptional states distinct from NK1 cells. Notably, longitudinal time comparisons quantify relative subtype proportions across sampling time points, whereas pseudotime analyses characterize cell‐state transition in cell lineage. Differential expression analysis showed that NK2 enriched genes involved in osteoclast differentiation, response to interleukin‐12 (IL‐12), and Th1 and Th2 cell differentiation (Figure 3D, and Table S6 in Supporting Information S3). IL‐12, primarily secreted by macrophages and DCs, induces T and NK cells to produce interferon‐γ (IFN‐γ), an anti‐osteoclastogenic factor that inhibits osteoclast formation [32]. These findings suggest that NK2 cells may participate in osteoclast regulation through IL‐12‐mediated signaling during treatment. To further investigate the potential role of NK2 cells in osteoclast regulation, we examined the expression dynamics of genes associated with osteoclast differentiation across study groups. In NK2 cells, genes involved in osteoclast differentiation were downregulated in the normal group and the early treatment stage (XLH_1.1) but upregulated in the late treatment stage (XLH_1.4) (Figure 3E), implying that elevated expression of these genes in NK2 cells may contribute to bone formation by suppressing osteoclast activity during treatment. Genes highly expressed in NK3 cells were also enriched in Th1, Th17, and Th2 cell differentiation (Figure 3D, and Table S6 in Supporting Information S3).

Next, we further investigated gene temporal expression patterns of NK2 and NK3 cells. Genes in NK2 and NK3 cells could be classified into four (C1–C4) and five (C1–C5) temporal expression patterns using Mfuzz (Figure S3D and S3E in Supporting Information S1, and Table S7 in Supporting Information S3). Genes in the C3 pattern of NK2 cells and C1 pattern of NK3 cells were enriched in T cell receptor signaling and lymphocyte differentiation pathways (Figure S3D and S3E in Supporting Information S1, and Table S7 in Supporting Information S3). These genes were highly expressed in the pre‐treatment group, downregulated after treatment, while expressed at low levels in the normal group (Figure S3D and S3E in Supporting Information S1, and Table S7 in Supporting Information S3). All these results suggest that there could have potential communication between NK and T cells during treatment, which may be related with disease recovery.

To further elucidate cellular communication between NK and T cells, we performed cellular communication analysis. Interactions between Th2 cells and NK subtypes were stronger in disease groups (XLH_0 and XLH_1.1–XLH_1.4) than in the normal group, with the most prominent interactions observed between Th2 and NK2 cells during treatment (Figure 3G). Th2–NK3 interactions were enhanced in the pre‐treatment group (XLH_0) and early treatment stages (XLH_1.1 and XLH_1.2) but weakened in the normal and the late stage (XLH_1.4) (Figure 3G). Interactions between NK cell subtypes and Th17 cells showed minimal variation across groups (Figure 3F). Ligand–receptor analysis revealed that NK2–Th2 primarily connected through ADRB2–VEGFB pairs in the pre‐treatment group (XLH_0) and the XLH_1.2 and XLH_1.3 stages, but disappeared in the normal group and the XLH_1.4 stage (Figure 3H). Across all groups, NK3–Th17 interactions were mediated by CCL4L2–VSIR pairs and Th17–NK3 interactions were mediated by TNFSF10–RIPK1 pairs, whereas Th17–NK3, Th2–NK2, and Th2–NK3 were connected through SELL–SELPLG ligand–receptor pairs throughout treatment (Figure 3H).

These findings reveal dynamic interactions between NK and T cell subtypes during treatment, with NK2 and NK3 cells potentially contributing to bone formation and disease recovery in XLH.

### 3.4 Functions of B Cells, Monocyte Subtypes, and Macrophages During Treatment

**FIGURE 4:**
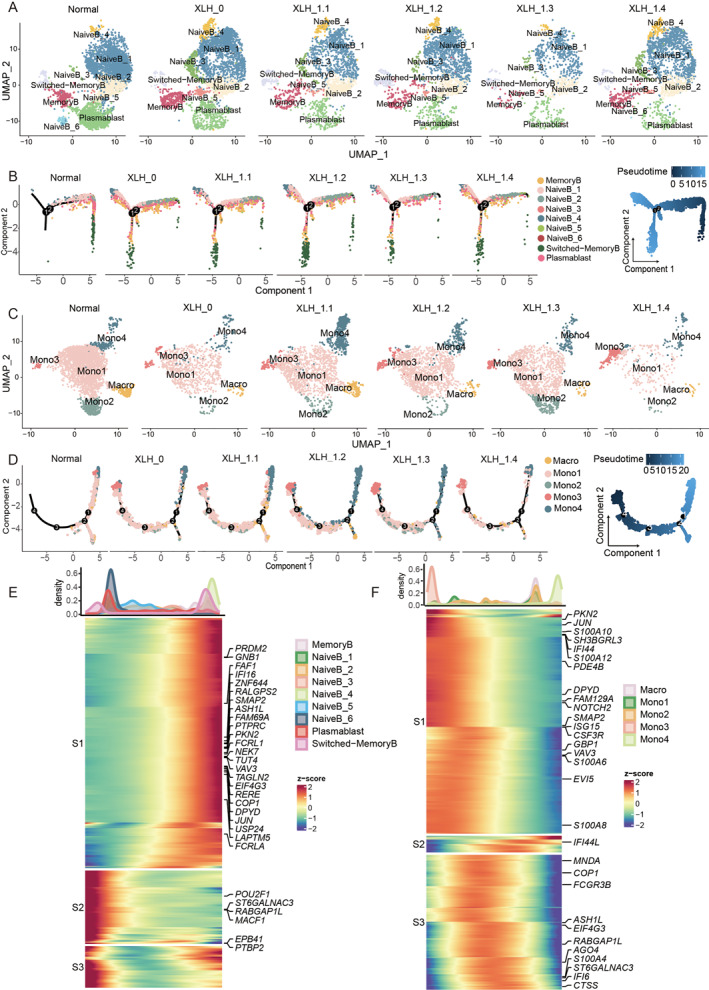
Subtype analysis of B cells, monocytes, and macrophages. (A) Uniform manifold approximation and projection (UMAP) plot of 13,984 B cells colored by cell subtype and split by group. (B) UMAP plot showing the pseudotime trajectory of B cell subtypes, split by group. (C) UMAP plot of 9,620 monocytes and macrophages in each group colored by cell subtype. (D) UMAP plot illustrating the pseudotime trajectory of monocyte subtypes and macrophages in each group analyzed by monocle2. (E) Heatmap showing gene expression profiles along the pseudotime trajectory of B cells. The color gradient represents expression levels. The top graph shows the density distribution of cell subtypes across pseudotime states. Terms S1–S3 represent gene modules identified based on similar gene expression dynamics along the pseudotime trajectory. Cells are ordered according to pseudotime, with early‐stage cell on the left and late‐stage cells on the right. (F) Heatmap showing gene expression profiles along the pseudotime trajectory of monocyte subtypes and macrophages. Macro, macrophage; MemoryB, memory B cell; Mono1–Mono4, monocyte subtypes; NaiveB_1–NaiveB_6, naive B cell subtypes; Switched‐MemoryB, switched memory B cell.

We next performed a subtype analysis of B cells, identifying 13,984 cells clustered into memory B (MemoryB) cells, plasmablasts, switched‐memory B (Switched‐MemoryB) cells, and six groups of naïve B cells (NaiveB_1–6) based on canonical markers (Figure 4A, and Figure S4A and S4B in Supporting Information S1). In the comparison across study groups, the proportion of each subtype varied across groups (Figure S4C in Supporting Information S1). Pseudotime analysis revealed that NaiveB_1 and Switched‐MemoryB cells were enriched toward the terminal region of the inferred trajectory, whereas MemoryB cells were mainly distributed toward the initial region (Figure 4B). A total of 9,186 monocytes and 434 macrophages were also identified and monocyte were classified into four subtypes (Mono1–4) (Figure 4C, Figure S4D and S4E in Supporting Information S1). The proportion of Mono3 increased from the Normal group through the treatment time points (XLH_0 and XLH_1.1–XLH_1.4), whereas other subsets exhibited fluctuating trends (Figure S4F in Supporting Information S1). Pseudotime analysis revealed that macrophages were predominantly distributed in intermediate pseudotime regions, whereas Mono3 and Mono2 were enriched toward the initial region of the inferred trajectory (Figure 4D). Mono3 and Mono1 were mainly observed in disease groups (XLH_0 and XLH1.1–XLH_1.4) and were largely absent in the normal group at the initial region of the pseudotime trajectory, consistent with condition‐associated shifts in myeloid transcriptional states.

The Monocle2 pseudotime heatmap highlighted genes with dynamic expression patterns along the inferred B‐cell transcriptional‐state continuum (Figure 4E). Specifically, *POU2F1* and *MACF1* showed higher expression in cells located toward the early pseudotime region, whereas *PRDM2* and *FCRLA* were enriched toward the late pseudotime region. Similarly, the pseudotime heatmap of the monocyte compartment and macrophage revealed genes varying along the inferred trajectory (Figure 4F), with *PKN2, JUN*, and *SH3BGRL3* showing higher expression toward early pseudotime, whereas *IFI44L* decreased toward late pseudotime. Mfuzz analysis revealed dynamic transcriptional changes in both B cells and monocyte/macrophage populations during treatment, and identified five temporal expression patterns in each cell population (C1–C5 for B cells and C1–C5 for monocytes/macrophages). In B cells, genes in C3 pattern were highly expressed during treatment but downregulated in the normal group and the late stage and were enriched in the neucleotide‐binding oligomerization domain (NOD)‐like receptor signaling pathway (Figure S4G in Supporting Information S1, and Table S8 in Supporting Information S3). Similarly, Genes in C1 pattern of monocytes and macrophages exhibited a similar trend, primarily associated with leukocyte transendothelial migration (Figure S4H in Supporting Information S1, and Table S9 in Supporting Information S3).

These results reveal dynamic transcriptional and compositional changes in B cells and monocyte/macrophage populations during treatment, indicating their potential roles in immune regulation and recovery in XLH.

### 3.5 Th2, Th17, Treg, and NK Cell Subtypes May Play Important Roles During XLH Treatment

**FIGURE 5:**
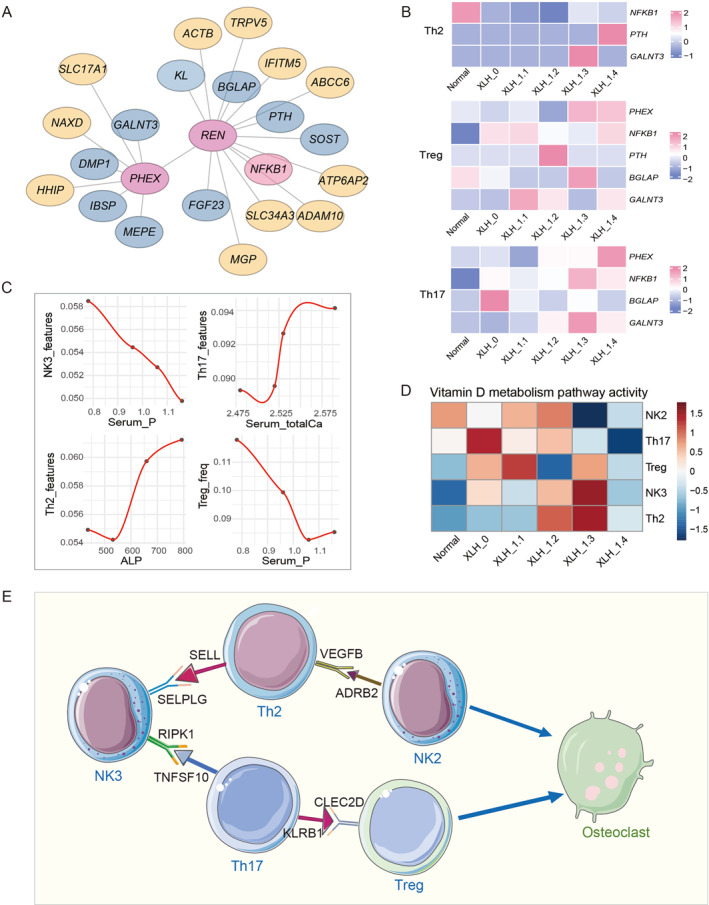
Further analysis of T helper 2 (Th2) cells, T helper 17 (Th17) cells, regulatory T (Treg) cells, and natural killer (NK) cells. (A) Protein‐protein interaction (PPI) network of *PHEX*, the pink nodes indicate the interaction path connecting *NFKB1* and *PHEX* through *REN*. Blue nodes represent genes related to X‐linked hypophosphatemia (XLH), whereas yellow nodes represent other genes in the PPI network. (B) Heatmap showing expression levels of genes related to XLH in *PHEX* PPI network in each group of T cell subtypes. (C) Nonparametric regression curve of clinical laboratory parameters and characteristics of cell subtypes. (D) Heatmap illustrating vitamin D metabolism pathway activity inferred by METAFlux of each cell subtype in each group. (E) Predicted schematic diagram of potential cellular interactions that could influence osteoclast growth and differentiation during XLH treatment. Cell‐cell communication analysis identified communication between NK3–Th2 (SELL–SELPLG and RIPK1–TNFSF10), Th2–NK2 (VEGFB–ADRB2), and Th17–Treg (CLEC2D–KLRB1) cells. Combined with temporal gene expression pattern, cell‐cell communication, and pathway enrichment analysis, these finding suggest that NK2 and Treg cells may participate in osteoclast regulation and bone remodeling during treatment. Blue arrows indicate proposed regulatory relationships. ALP, alkaline phosphatase; NK2–NK3, natural killer cell subtypes 2–3; NK3_features, NK3 signature activity score; Serum_P, serum phosphorus; Serum_totalCa, serum total calcium; Th2_features, Th2 signature activity score; Th17_features, Th17 signature activity score; Treg_freq, Treg cell frequency.

To further elucidate the functions of Th2, Th17, Treg, NK2, and NK3 cells during treatment, we performed integrative analyses combining transcriptional, interaction, and metabolic data. A minimum spanning tree of the *PHEX*‐related PPI network was constructed (Figure 5A). *NFKB1*, which was highly expressed in Th17 cells across disease groups (XLH_0 and XLH_1.1–XLH_1.4) (Figure 2F), was functionally linked to *PHEX* through *REN* (Figure 5A). *PHEX*‐associated genes from the STRING PPI network revealed differential expression patterns across T cell subtypes (Figure 5B). *NFKB1, PTH*, and *GALNT3* were downregulated in Th2 cells across most groups (Figure 5B), whereas in Treg cells, the expression of *NFKB1* increased in the pre‐treatment group (XLH_0), decreased during treatment, and elevated again in the late stage (XLH_1.4). In Th17 cells, *PHEX*, *NFKB1*, and *GALNT3* were all upregulated at late treatment stages (XLH_1.3 and XLH_1.4) (Figure 5B).

To explore whether clinical response–related biochemical indicators are associated with immune‐cell programs, we assessed the relationship between longitudinal clinical laboratory parameters and immune‐cell characteristics (Figure 5C, Figure S6 in Supporting Information S1). Serum phosphorus, a key biochemical marker of XLH and a primary indicator used to monitor burosumab response, was analyzed together with other routine parameters, including serum calcium and alkaline phosphatase (ALP), which reflect mineral homeostasis and bone turnover, respectively. Serum phosphate increased from 0.78 to 1.16 mmol/L after burosumab therapy and remained above baseline thereafter, whereas ALP decreased steadily from 792 to 436 U/L (Table 1) during treatment, supporting a treatment response.

**TABLE 1:** Clinical information of patients with X‐linked hypophosphatemia.

Variable	Patient 1	Patient 2	Patient 2—Week 2	Patient 2—Week 4	Patient 2—Week 6
	XLH_0	XLH_1.1	XLH_1.2	XLH_1.3	XLH_1.4
Gender	Male	Male			
Age (months)	15	13			
Treatment	None	Burosumab 10 mg, SC, q2w			
*PHEX* mutation type	Missense	None			
Mutation description	c. 22226492C > T	None			
Clinical experimental results					
Vitamin D (ng/mL)		30.92	33.45	27.23	38.34
Total serum calcium (mmol/L)		2.53	2.59	2.48	2.52
Serum phosphate (mmol/L)		0.78	1.16	1.06	0.96
Alkaline phosphatase (U/L)		792	659	531	436
Parathyroid hormone (pg/mL)		115	—	112	68.2
Urinary phosphorus (mmol/L)		29.3	—	59.2	51.8
Urinary calcium (mmol/L)		0.71	—	0.09	0.26

For immune features, we defined cell subtype–specific gene signatures using the top 20 highly expressed genes in each cell subtype and quantified their activity at the single‐cell level using AUCell; scores were then summarized at the group level. We applied LOESS regression as an exploratory approach to visualize nonlinear associations between clinical indicators, immune feature scores, and immune cell proportions. Notably, serum phosphorus (Serum_P) showed a negative association with NK3 feature activity (NK3_features) and with Treg cell frequency (Treg_freq), whereas Th17 feature activity (Th17_features) was positively associated with serum calcium (Serum_totalCa), and Th2 feature activity (Th2_features) was positively associated with ALP levels (Figure 5C). Additional associations are shown in Figure S6 in Supporting Information S1. These results suggest that clinical biochemical status is accompanied by coordinated shifts in specific immune‐cell programs, providing clinical context for the observed immune remodeling. Vitamin D metabolism pathway is closely related to bone formation; therefore, we performed metabolic flux analysis using METAFlux to investigate its association with T and NK cell subtypes (Figure 5D). This pathway revealed decreased activity at the late treatment late stage (XLH_1.4) across all subtypes (Figure 5D). Notably, vitamin D fluxes were elevated during early treatment stages (XLH_1.1 and XLH_1.2) in NK2 and Th17 cells, and increased at the late stage (XLH_1.3) in NK3 and Th2 cells, suggesting stage‐specific association with vitamin D metabolism (Figure 5D). Moreover, NK‐CD8^+^ T cell interactions were stronger in the pre‐treatment group (XLH_0) and the late treatment stage (XLH_1.4) compared with the normal group and the early treatment stage (XLH_1.1) (Figure S5 in Supporting Information S1), implying their potential involvement in disease modulation.

Based on these findings, we suggest a potential cellular interaction network influencing osteoclast regulation and disease prognosis during XLH treatment (Figure 5E). These intercellular connections suggest that NK2 and Treg cells may modulate osteoclast differentiation, potentially contributing to disease improvement during burosumab therapy.

## 4 Discussion

XLH is caused by mutations in the *PHEX* gene, leading to elevated *FGF23* levels and hypophosphatemia [33]. *PHEX*, primarily expressed in osteocytes and osteoblasts, regulates *FGF23* through degradation of specific peptide substrates and glycoproteins [34]. Mutations in *PHEX* upregulate *FGF23* by increasing acid serine‐ and aspartate‐rich motif (ASARM) peptides and osteopontin storage, inhibiting local bone mineralization [35]. Elevated *FGF23* inhibits the production of 1, 25‐(OH)_2_D_3_, impairs phosphate reabsorption, and reduces serum phosphorus [36]. Burosumab, a monoclonal antibody targeting *FGF23*, has shown promise in treating XLH. However, its potential immunological effects, including recurrent infections, require further investigation. In this context, our findings indicate that burosumab treatment may be associated with alterations in immune cell functions, highlighting the need for further investigation in larger and longitudinal cohorts.

Genes associated with XLH, including *PTH, MEPE, FGF23, DMP1, NFKB1*, and *BGLAP*, were identified in the *PHEX* PPI network. *NFKB1* was upregulated in Th17 cells in disease groups and involved in Th1 and Th2 differentiation (Figure 2F). *RANKL* activation of *TRAF6* leads to *NFKB1* activation, promoting osteoclast differentiation [37]. Proinflammatory cytokines, including tumor necrosis factor‐α (TNF‐α) and interleukin‐1β, activate *RANKL‐*mediated osteoclastogenesis. [38, 39]. Notably, TNF‐α can also be secreted by NK cells. Previous studies provide indirect support for our hypothesis that NK cells could influence *NFKB1* expression in Th17 cells through TNF‐α secretion, thereby impacting osteoclast activity during XLH treatment.

1, 25‐(OH)_2_D_3_, essential for immune function, also regulates NK cell activity by decreasing IFN‐γ and cytotoxic markers [40, 41]. Kitajima's study from 1989 found NK cell activity was lower in hypophosphatemic rickets patients but increased with vitamin D treatment [42]. Our results indicate that NK2 cells, highly expressed genes enriched in osteoclast differentiation during treatment stages, and that NK2 exhibited higher vitamin D metabolism activity scores in early treatment stages. Consistent with previous studies, our research provides a deeper understanding of the role of NK cells in hypophosphatemic rickets.

1, 25‐(OH)_2_D_3_ promotes Treg cell differentiation by increasing interleukin‐10 (IL‐10) and inhibiting interleukin‐6, which in turn regulates Th17 cell differentiation [43]. Th17 cells also promote *RANKL* expression by producing interleukin‐17, thereby inducing osteoclast formation [28]. In patients with rheumatoid arthritis and multiple myeloma, the proportion of Th17 cells increased [28], indicating that Th17 cells could be involved in bone‐related disease. Our findings highlight dynamic interactions among Th17, Th2, and Treg cells during treatment, suggesting that Th17 cells may contribute to immune regulation and bone metabolic processes during XLH treatment.

Treg cells inhibit osteoclast formation through secreting IL‐10 and transforming growth factor‐β1 [28]. In our study, genes related to osteoclast differentiation were upregulated in Treg cells during the early treatment stage and returned to normal levels in the late treatment stage, hinting that Treg cells could affect osteoclast differentiation in the early treatment stage. Genes enriched in the sphingolipid signaling pathway in Treg cells also exhibited a similar trend. Sphingosine and its derivatives regulate osteoblast functions [44]. Especially, sphingomyelinase‐3 deficiency in mice disrupts cartilage and bone matrix, causing severe skeletal deformities [45]. Overall, these studies support our hypothesis that Treg cells may involve in osteogenesis through sphingolipid pathway during treatment.

Several limitations should be noted. First, this study is based on a single case observed longitudinally which limits the generalizability of the findings. Therefore, the observed cellular dynamics should be interpreted as descriptive and hypothesis‐generating rather than inferential. However, given the ultrarare nature of XLH and the challenges of longitudinal single‐cell profiling, this study provides valuable exploratory insights into immune alternation of XLH during burosumab treatment. Second, healthy control data were obtained from a public dataset rather than collected contemporaneously, which may introduce batch effects despite computational correction. Third, the analysis was restricted to peripheral blood and may not fully reflect immune processes within the bone microenvironment where XLH pathology primarily occurs. Future studies with larger cohorts and bone tissue profiling will be needed to validate and extend these findings.

## 5 Conclusion

Our study presents the first longitudinal single‐cell transcriptomic characterization of peripheral blood immune cells in pediatric patients with XLH undergoing burosumab therapy. By analyzing over 93,000 single cells across healthy controls to multiple treatment stages, we identified dynamic alterations in T cell and NK cell subtypes that may be relevant to bone metabolism regulation. Furthermore, correlations between immune features and serum biochemical markers as well as alterations in vitamin D metabolic flux, highlight immune–metabolic crosstalk during treatment. Collectively, these findings provide novel insights into the immunological mechanisms underlying XLH during burosumab treatment and identify potential cellular and molecular targets for optimizing therapeutic strategies.

## Author Contributions

**Yue Xie:** bioinformatics and data analysis, writing – original draft preparation, writing – review and editing. **Li Li:** writing – original draft preparation, writing – review and editing. **Rong Li:** conceptualization, writing – review and editing. **Yihong Sun:** bioinformatics and data analysis, writing – review and editing. **Ting Zhou:** writing – original draft preparation, writing – review and editing. **Yupeng Cun:** conceptualization, bioinformatics and data analysis, investigation, writing – original draft preparation, writing – review and editing. **Gaohui Zhu:** conceptualization, investigation, writing – original draft preparation, writing – review and editing.

## Funding

The authors have nothing to report.

## Ethics Statement

The study was approved by the Ethics Committee of Children's Hospital of Chongqing Medical University (No. 2022340) and was conducted according to the guidelines of the Declaration of Helsinki.

## Consent

The written informed consents were obtained from the patient’s parents.

## Conflicts of Interest

The authors declare no conflicts of interest.

## Supporting information


Supporting Information S1

Supporting Information S2

Supporting Information S3

## Data Availability

The scRNAseq and WGS data are available at the National Genomics Data Center with BioProject ID PRJCA041716 and HRA017909 (https://ngdc.cncb.ac.cn/gsub/submit/bioproject/subPRO061378/overview).
