# Supplementary material for: Longitudinal Single‐Cell Transcriptomic Profiling Reveals Dynamic Immune Cell Alterations During Burosumab Therapy in X‐Linked Hypophosphatemia
Source: Pediatr Discov. 2026 Jun 27;4(2):e70053. doi: 10.1002/pdi3.70053 (PMC13320813; doi:10.1002/pdi3.70053)
Supplement: Supplementary file 1 — Supporting Information S1 [file PDI3-4-e70053-s003.pdf]

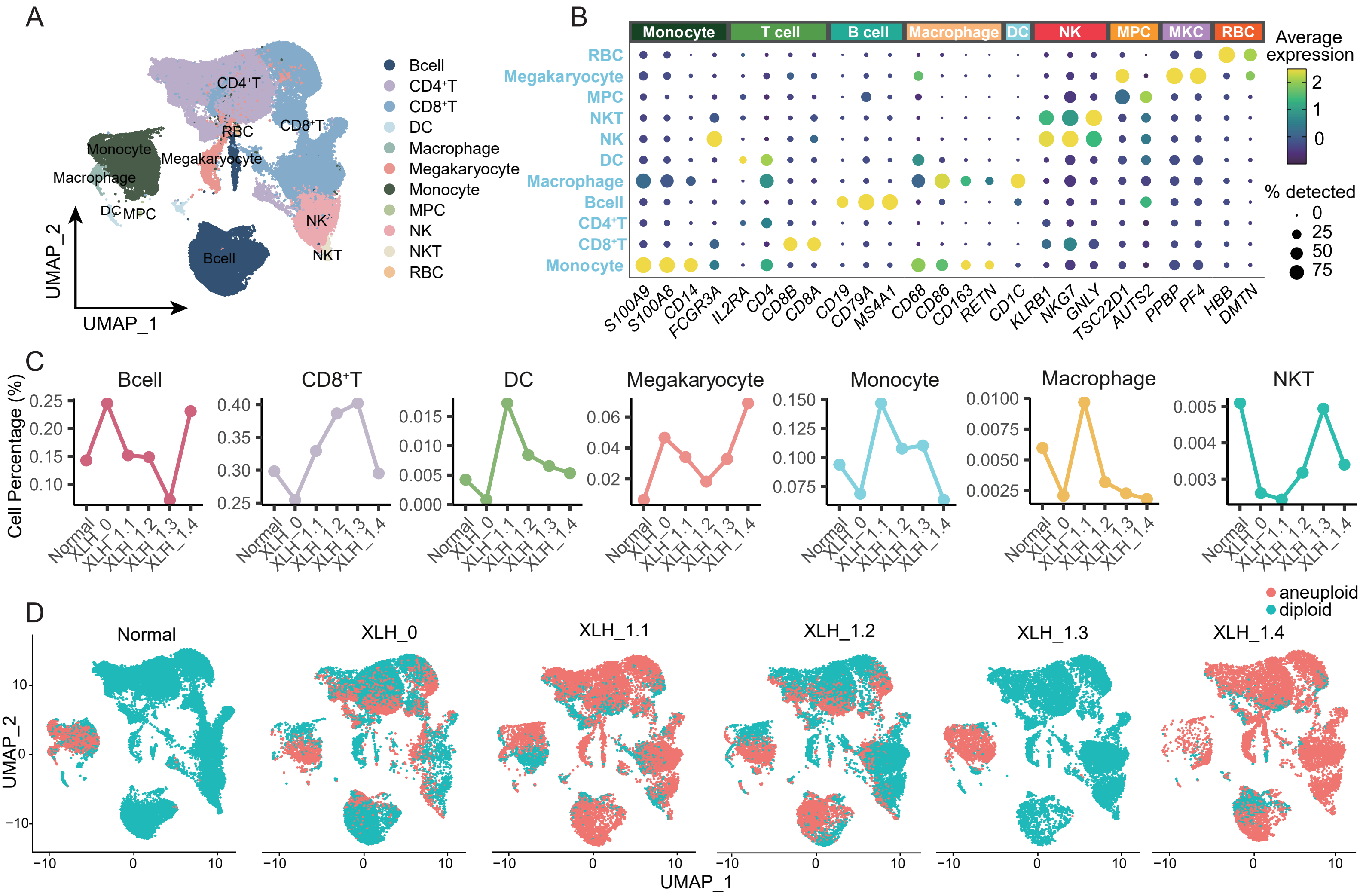

Figure S1. Annotation of peripheral blood mononuclear cells from patients with X-linked hypophosphatemia (XLH).

A. Uniform manifold approximation and projection (UMAP) plot of cells colored by cell type.

B. Dot plot displaying the expression level of canonical marker genes in each cell type. The color represents gene expression level, the dot size represents the percentage of cells expressing the gene in each cell type.

C. Line graph showing changes in the percentage of different cell types across study groups.

D. UMAP plot showing copy number variation patterns across study groups.

Normal represents healty controls, XLH_0 denotes the pre-treatment sample from one patient with XLH, whereas XLH_1.1–XLH_1.4 denote longitudinal samples collected from the second patient with XLH during burosumab treatment.

MPC, multipotent progenitor cell; MKC, megakaryocyte; DC, dendritic cell; NKT, natural killer T cell; NK, natural killer cell; RBC, red blood cell; CD8⁺T, CD8⁺T cell; CD4⁺T, CD4⁺T cell; Bcell, B cell.

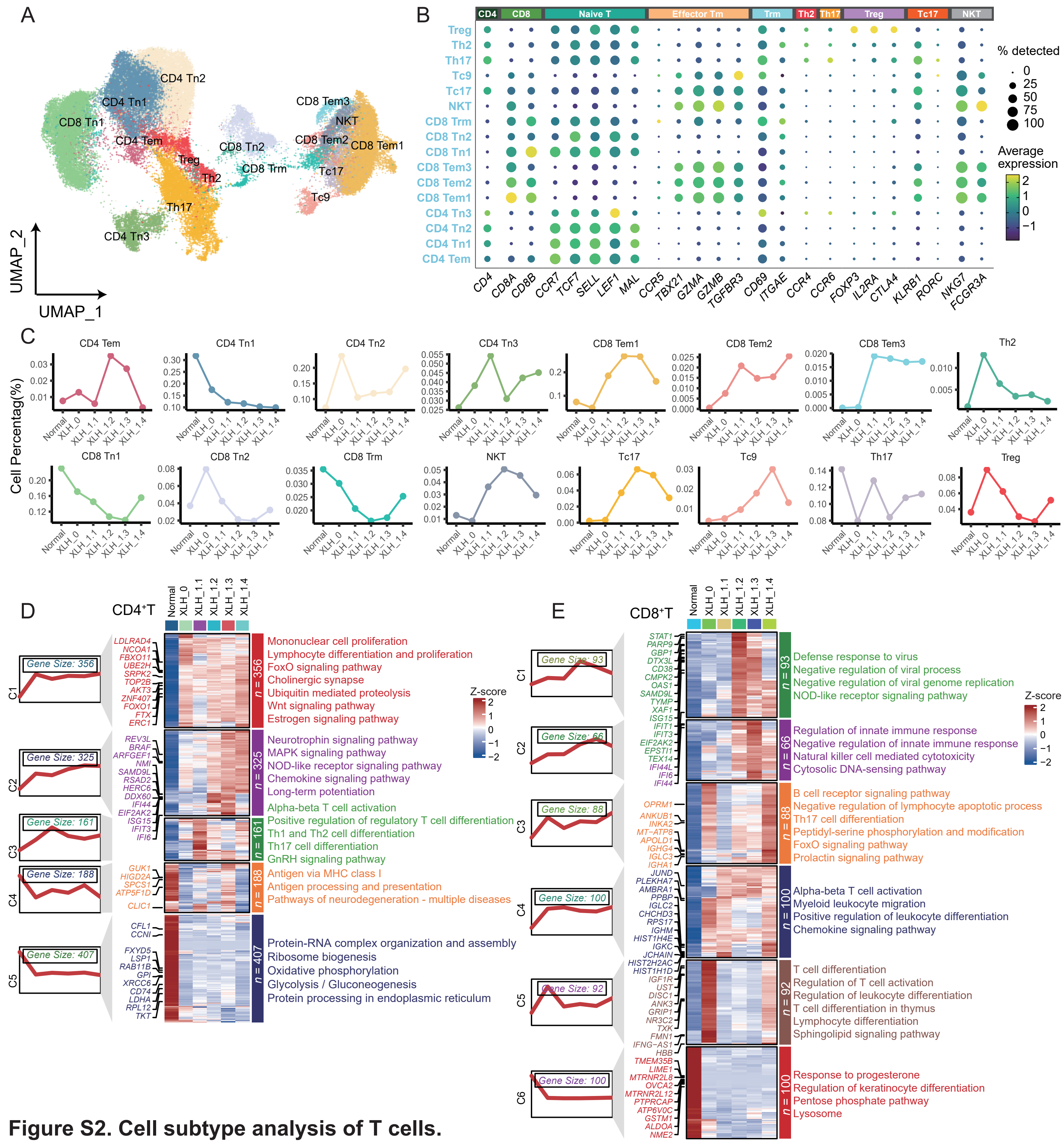

Figure S2. Cell subtype analysis of T cells.

A. Uniform manifold approximation and projection plot of T cell subtypes colored by cell subtypes.

B. Dot plot showing the expression of canonical marker genes in T-cell subtypes. The color represents gene expression level, the dot size represents the percentage of cells expressing the gene in each cell subtype.

C. Line graph showing changes in the percentage of different groups within each T-cell subtype.

D. Heatmap showing genes and Gene Ontology (GO) terms and Kyoto Encyclopedia of Genes and Genomes (KEGG) pathways of CD4$^+$ T cells in different temporal gene expression patterns over study groups. The line graph on the left shows trends over study groups. The number shown in each panel (Gene Size) indicates the number of genes assigned to the corresponding temporal expression pattern.

E. Heatmap showing genes and GO terms and KEGG pathways of CD8$^+$T cells in different temporal gene expression patterns over study groups.

CD4 Tn1-CD4 Tn3, naive CD4$^+$T-cell subtypes; CD4 Tem, memory CD4$^+$T cell; CD8 Tem1-CD8 Tem3, memory CD8$^+$T-cell subtypes; CD8 Tn1-CD8 Tn2, naive CD8$^+$T-cell subtypes; CD8 Trm, CD8$^+$ tissue-resident memory T cell; Treg, regulatory T cell; NKT, natural killer T cell; NOD, nucleotide-binding olgomerization domain; MAPK, mitogen-activated protein kinase; GnRH, gonadotropin-releasing hormone.

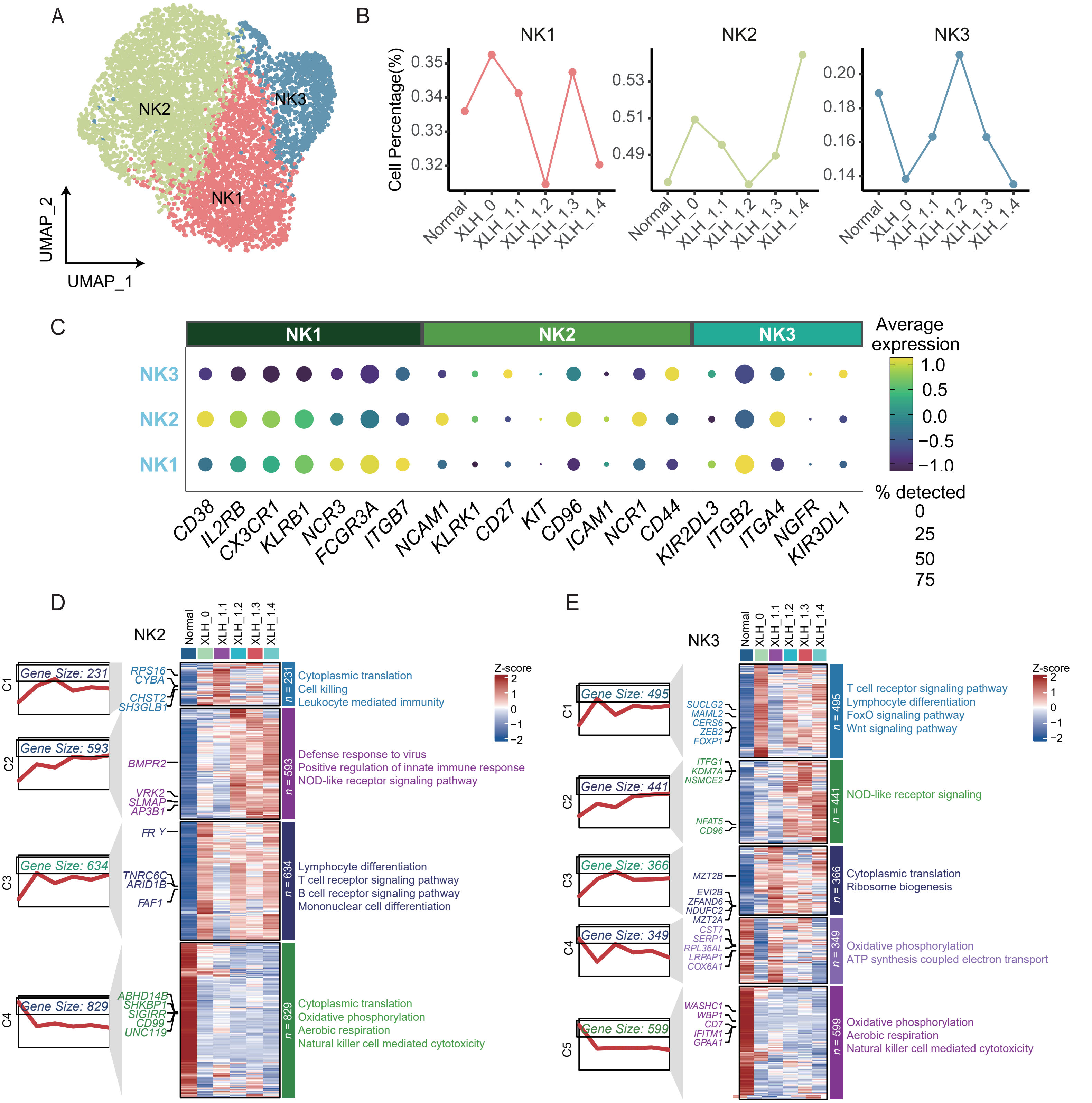

Figure S3. Cell subtype analysis of natural killer (NK) cells.

A. Uniform manifold approximation and projection plot of NK cells colored by cell subtypes.

B. Line graph showing changes in the percentage of different study groups within each NK cell subtype.

C. Dot plot displaying the expression of marker genes in NK cell subtypes. The color represents gene expression, the dot size represents the percentage of cells expressing the gene in each cell subtype.

D. Heatmap showing genes and relevant Kyoto Encyclopedia of Genes and Genome (KEGG) pathways and Gene Ontology (GO) terms in different temporal gene expression patterns over study groups of NK2 cells. The line graph on the left shows trends over different study groups. The number shown in each panel (Gene Size) indicates the number of genes assigned to the corresponding temporal expression pattern.

E. Heatmap showing genes and relevant KEGG pathways and GO terms in different temporal gene expression patterns over study groups of NK3 cells.

NK1–NK3, natural killer cell subtypes 1–3; NOD, nucleotide-binding oligomerization Domain.

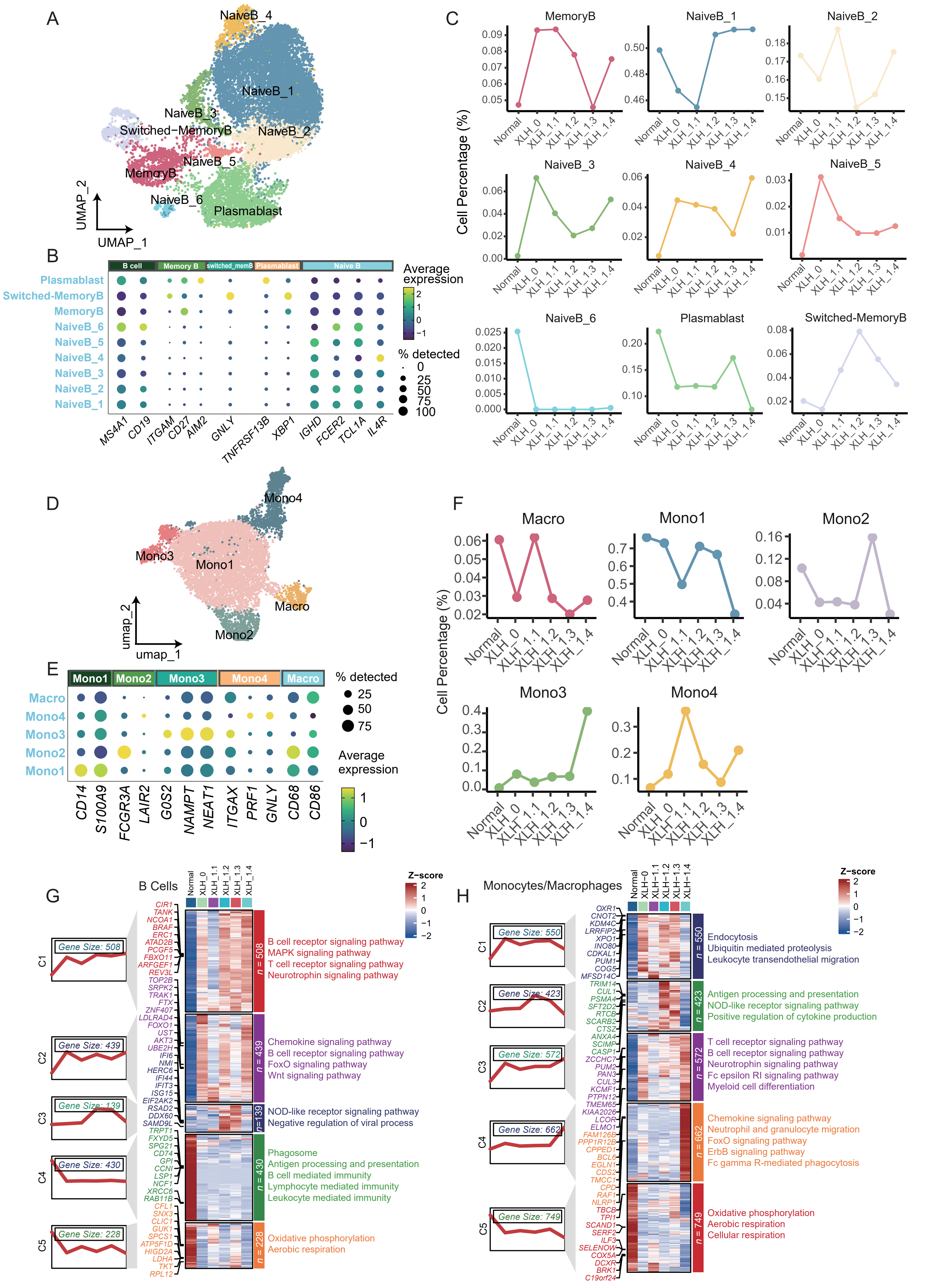

A
NaiveB_4
NaiveB_1
NaiveB_3
Switched−MemoryB
NaiveB_2
NaiveB_5
MemoryB
NaiveB_6
Plasmablast
UMAP_2
UMAP_1
B
B cell
Memory B
switched_memB
Plasmablast
Naive B
Plasmablast
Switched-MemoryB
MemoryB
NaiveB_6
NaiveB_5
NaiveB_4
NaiveB_3
NaiveB_2
NaiveB_1
MS4A1
CD19
ITGAM
CD27
AIM2
GNLY
TNFRSF13B
XBP1
IGHD
FCER2
TCL1A
IL4R
Average expression
% detected
C
Cell Percentage (%)
MemoryB
NaiveB_1
NaiveB_2
NaiveB_3
NaiveB_4
NaiveB_5
NaiveB_6
Plasmablast
Switched-MemoryB
Normal
XLH_0
XLH_1.1
XLH_1.2
XLH_1.3
XLH_1.4
D
Mono4
Mono3
Mono1
Macro
Mono2
umap_2
umap_1
E
Mono1
Mono2
Mono3
Mono4
Macro
CD14
S100A9
FCGR3A
LAIR2
G0S2
NAMPT
NEAT1
ITGAX
PRF1
GNLY
CD68
CD86
% detected
Average expression
F
Cell Percentage (%)
Macro
Mono1
Mono2
Mono3
Mono4
G
B Cells
Z−score
C1
Gene Size: 508
C2
Gene Size: 439
C3
Gene Size: 139
C4
Gene Size: 430
C5
Gene Size: 228
n = 508
B cell receptor signaling pathway
MAPK signaling pathway
T cell receptor signaling pathway
Neurotrophin signaling pathway
n = 439
Chemokine signaling pathway
B cell receptor signaling pathway
FoxO signaling pathway
Wnt signaling pathway
n=139
NOD-like receptor signaling pathway
Negative regulation of viral process
n = 430
Phagosome
Antigen processing and presentation
B cell mediated immunity
Lymphocyte mediated immunity
Leukocyte mediated immunity
n = 228
Oxidative phosphorylation
Aerobic respiration
H
Monocytes/Macrophages
Z−score
C1
Gene Size: 550
C2
Gene Size: 423
C3
Gene Size: 572
C4
Gene Size: 662
C5
Gene Size: 749
n = 550
Endocytosis
Ubiquitin mediated proteolysis
Leukocyte transendothelial migration
n = 423
Antigen processing and presentation
NOD-like receptor signaling pathway
Positive regulation of cytokine production
n = 572
T cell receptor signaling pathway
B cell receptor signaling pathway
Neurotrophin signaling pathway
Fc epsilon RI signaling pathway
Myeloid cell differentiation
n = 662
Chemokine signaling pathway
Neutrophil and granulocyte migration
FoxO signaling pathway
ErbB signaling pathway
Fc gamma R-mediated phagocytosis
n = 749
Oxidative phosphorylation
Aerobic respiration
Cellular respiration

Figure S4. Cell subtype analysis of B cells, monocytes, and macrophages.
A. Uniform manifold approximation and projection (UMAP) plot of B cells colored by cell subtypes.
B. Dot plot displaying the expression of marker genes in B cell subtypes. The color represents gene expression, and the dot size represents the percentage of cells expressing the gene in each cell subtype.
C. Line graph showing the percentage of each B cell subtype across study groups.
D. UMAP plot of monocyte subtypes and macrophages colored by cell types.
E. Dot plot displaying the expression of marker genes related to monocyte subtypes and macrophages.
F. Line graph showing the percentage of each monocyte subtype and macrophage across study groups.
G. Heatmap showing genes and relevant Kyoto Encyclopedia of Genes and Genomes (KEGG) pathways and Gene Ontology (GO) terms in different temporal gene expression patterns over study groups of B cells. The line graph on the left shows trends over study groups. The number shown in each panel (Gene Size) indicates the number of genes assigned to the corresponding temporal expression pattern.
H. Heatmap showing genes and relevant KEGG pathways and GO terms in different temporal gene expression patterns over study groups of monocytes and macrophages.
NaiveB_1–NaiveB_6, naive B-cell subtypes; MemoryB, memory B cell; Switched-MemoryB, switched memory B cell; Mono1–Mono4, monocyte subtypes; Macro, macrophage; NOD, nucleotide-binding olgomerization domain; MAPK, mitogen-activated protein kinase.

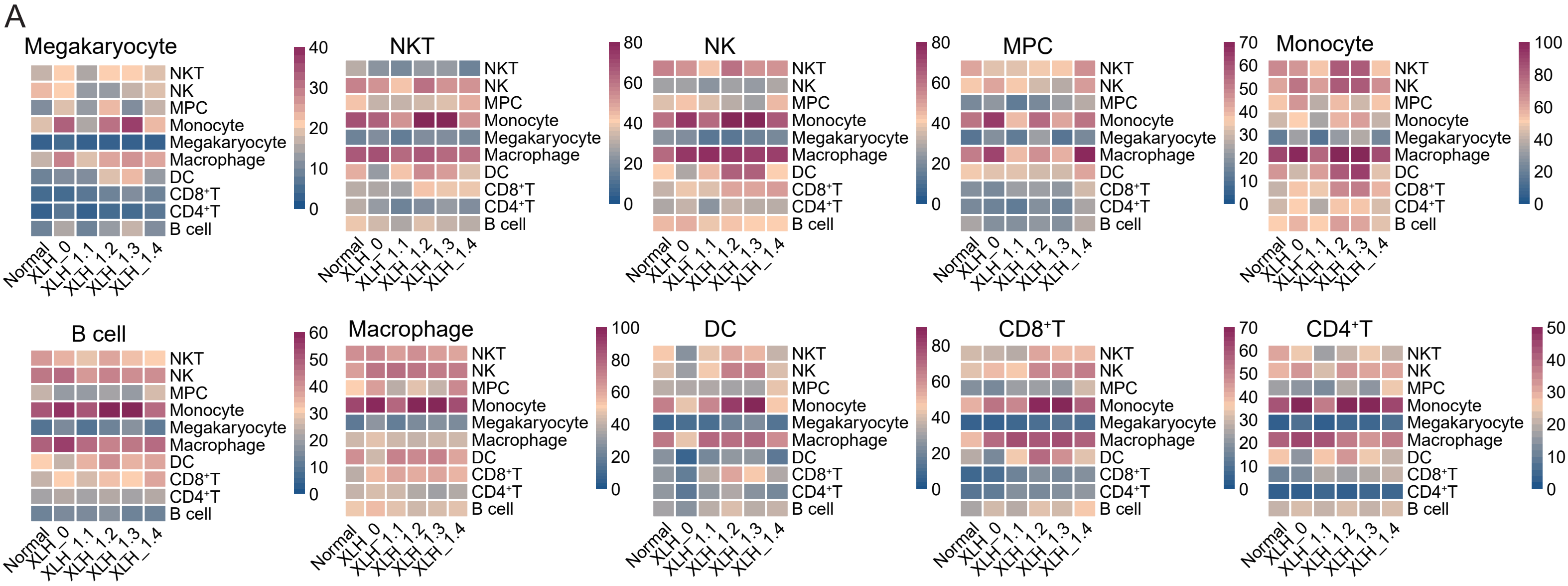

Figure S5. Cell-cell interactions among different cell types.

A. Heatmap showing cell-cell communication numbers of each cell type among each study group.

MPC, multipotent progenitor cell; DC, dendritic cell; NKT, natural killer T cell; NK, natural killer cell; CD4⁺T, CD4⁺T cell; CD8⁺T, CD8⁺T cell.

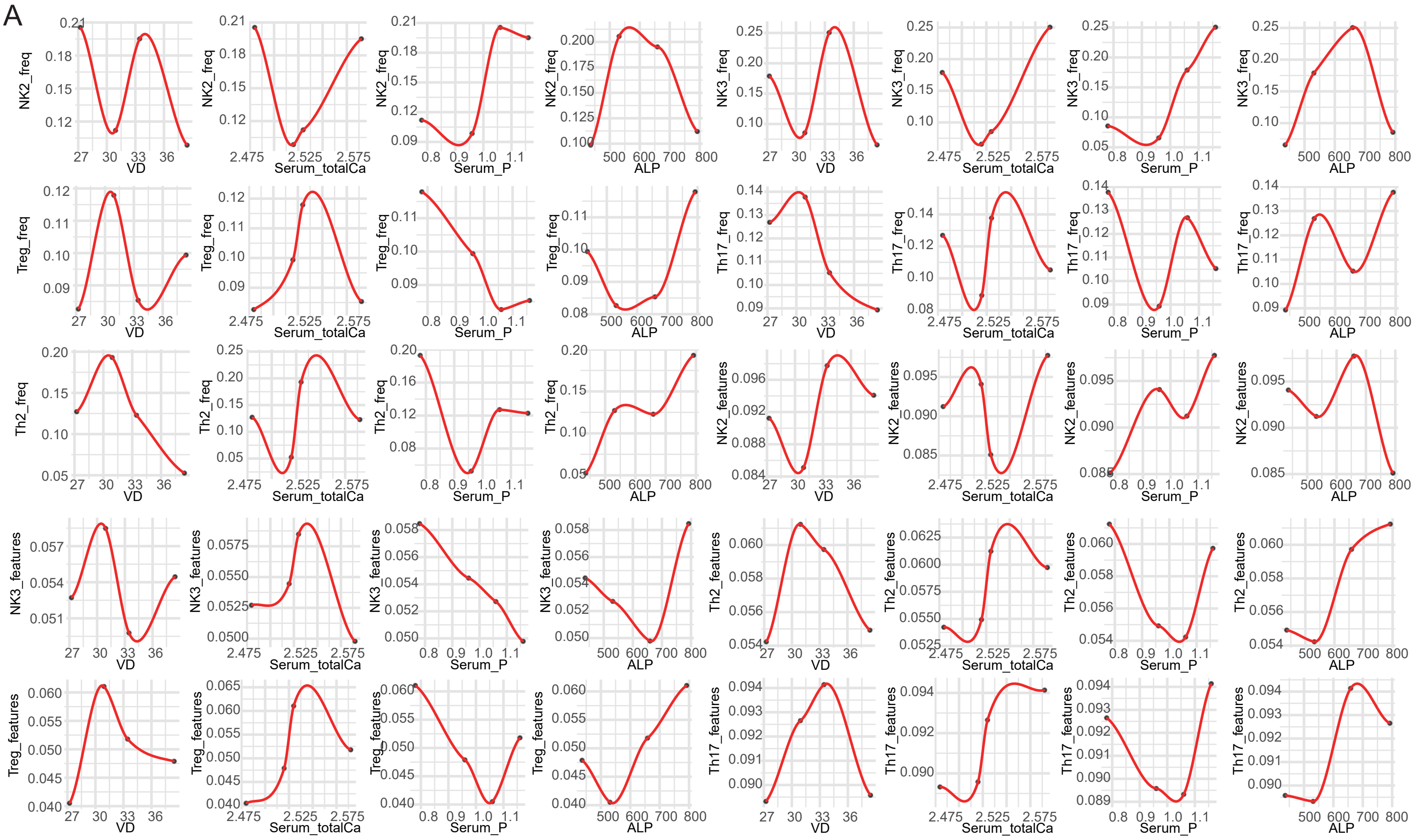

Figure S6. Non-parametric regression analysis of clinical parameters, cell activty scores, and cell proportions.

A. Non-parametric regression analysis of clinical laboratory parameters, activity scores, and proportions of T helper 2 (Th2) cells, T helper 17 (Th17) cells, regularoty T (Treg) cells, and natural killer cell subtypes 2-3 (NK2 and NK3).

VD, vitamin D; ALP, alkaline phosphatase; Serum_P, serum phosphorus; Serum_totalCa, serum total calcium; NK2_freq, frequency of NK2 cells; NK3_freq, frequency of NK3 cells; Th17_freq, frequency of Th17 cells; Th2_freq, frequency of Th2 cells; Treg_freq, frequency of Treg cells; NK2_features, NK2 gene signature activity score; NK3_features, NK3 gene signature activity score; Th17_features, Th17 gene signature activity score; Th2_features, Th2 gene signature activity score; Treg_features, Treg gene signature activity score.
