# Supplementary material for: Longitudinal Single‐Cell Transcriptomic Profiling Reveals Dynamic Immune Cell Alterations During Burosumab Therapy in X‐Linked Hypophosphatemia
Source: Pediatr Discov. 2026 Jun 27;4(2):e70053. doi: 10.1002/pdi3.70053 (PMC13320813; doi:10.1002/pdi3.70053)
Supplement: Supplementary file 2 — Supporting Information S2 [file PDI3-4-e70053-s002.docx]

**Captions of Supplementary Tables**

**Table S1.** **Overlapping differentially expressed genes (DEGs) identified in Pre_treatment *vs.* Normal and Treatment_stage *vs.* Pre_treatment comparisons.**

The table lists DEGs identified in both comparisons, including gene symbols, log2 fold changes, and adjusted *P* values. Pre_treatment corresponds to XLH_0, whereas Treatment_stage corresponds to XLH_1.3-XLH_1.4. Normal represents healthy controls. XLH_0 denotes the pre-treatment sample from one patient with XLH, whereas XLH_1.1-XLH_1.4 denote longitudinal samples collected from the second patient with XLH during burosumab treatment. XLH, X-linked hypophosphatemia.

**Table S2. Mfuzz-derived temporal gene expression patterns, corresponding gene expression profiles, and enriched Kyoto Encyclopedia of Genes and Genomes (KEGG) pathways in all cells.**

Genes were grouped into temporal expression patterns using Mfuzz (C1-C5). Pattern assignments, membership scores, temporal expression trends across treatment groups and significant KEGG enrichment results are provided.

**Table S3. Gene Ontology (GO) and Kyoto Encyclopedia of Genes and Genomes (KEGG) enrichment analyses of Mfuzz-derived temporal gene expression patterns in regulatory T cells.**

Genes were grouped into temporal expression patterns (C1-C5) using Mfuzz. GO and KEGG enrichment analyses were conducted for each pattern, and significantly enriched GO terms and KEGG pathways are listed.

**Table S4. Kyoto Encyclopedia of Genes and Genomes (KEGG) enrichment analyses of Mfuzz-derived temporal gene expression patterns in T helper 17 cells.**

Genes were grouped into temporal expression patterns (C1-C6) using Mfuzz. KEGG enrichment analyses were conducted for each pattern, and significantly enriched KEGG pathways are listed.

**Table S5. Gene Ontology (GO) and Kyoto Encyclopedia of Genes and Genomes (KEGG) enrichment analyses of Mfuzz-derived temporal gene expression patterns in CD4⁺ and CD8⁺ T cells.**
Genes were grouped into temporal expression patterns using Mfuzz, resulting in five patterns (C1-C5) in CD4^+^T cells and six patterns (C1-C6) in CD8^+^T cells. GO and KEGG enrichment analyses were conducted for each pattern, and significantly enriched GO terms and KEGG pathways are listed.

**Table S6. Kyoto Encyclopedia of Genes and Genomes (KEGG) enrichment analyses of differentially expressed genes (DEGs) in natural killer (NK) cell subtypes.**
DEGs identified in each NK cell subtype were subjected to KEGG enrichment analysis. The table presents significantly enriched KEGG pathways and their corresponding enrichment statistics.

**Table S7. Gene Ontology (GO) and Kyoto Encyclopedia of Genes and Genomes (KEGG) enrichment analyses of Mfuzz-derived temporal gene expression patterns in natural killer cell subtypes 2 and 3 (NK2 and NK3).**

Genes were grouped into temporal expression patterns using Mfuzz, resulting in four patterns (C1-C4) in NK2 cells and five patterns (C1-C5) in NK3 cells. GO and KEGG enrichment analyses were conducted for each pattern, and significantly enriched GO terms and KEGG pathways are listed.

**Table S8.** **Gene Ontology (GO) and Kyoto Encyclopedia of Genes and Genomes (KEGG) enrichment analyses of Mfuzz-derived temporal gene expression patterns in B cells.**

Genes were grouped into temporal expression patterns (C1-C5) using Mfuzz. GO and KEGG enrichment analyses were conducted for each pattern, and significantly enriched GO terms and KEGG pathways are listed.

**Table S9. Gene Ontology (GO) and Kyoto Encyclopedia of Genes and Genomes (KEGG) enrichment analyses of Mfuzz-derived temporal gene expression patterns in monocytes and macrophages.**

Genes were grouped into temporal expression patterns (C1-C5) using Mfuzz. GO and KEGG enrichment analyses were conducted for each pattern, and significantly enriched GO terms and KEGG pathways are listed.
